# Access to primary health care among women: the role of Ghana’s community-based health planning and services policy

**DOI:** 10.1017/S1463423619000185

**Published:** 2019-06-26

**Authors:** Joseph Asumah Braimah, Yujiro Sano, Kilian Nasung Atuoye, Isaac Luginaah

**Affiliations:** 1 Department of Geography and Planning, Queen’s University, Mackintosh-Corry Hall, Room E208, Kingston, Ontario, Canada; 2 Department of Sociology, Social Science Centre, University of Western Ontario, 1151 Richmond Street, London, Ontario, Canada; 3 Department of Geography, Social Science Centre, University of Western Ontario, 1151 Richmond Street, London, Ontario, Canada

**Keywords:** Community-based Health Planning and Services, Ghana, health care access, primary health care, Upper West Region, women

## Abstract

**Background:**

Ghana in 1999 adopted the Community-based Health Planning and Service (CHPS) policy to enhance access to primary health care (PHC) service. After two decades of implementation, there remains a considerable proportion of the country’s population, especially women who lack access to basic health care services.

**Aim:**

The aim of this paper is to understand the contribution of Ghana’s CHPS policy to women’s access to PHC services in the Upper West Region (UWR) of Ghana.

**Methods:**

A logistic regression technique was employed to analyse cross-sectional data collected among women (805) from the UWR.

**Findings:**

We found that women who resided in CHPS zones (OR = 1.612; *P ≤* 0.01) were more likely to have access to health care compared with their counterparts who resided in non-CHPS zones. Also, rural-urban residence, distance to health facility, household wealth status and marital status predicted access to health care among women in the region. Our findings underscore the need to expand the CHPS policy to cover many areas in the country, especially rural communities and other deprived localities in urban settings.

## Introduction

Although access to health care is a fundamental human right, a considerable number of people, especially those in the low- and middle-income countries, lack access to this essential service (World Health Organization, 2016). Indeed, several developmental meetings and agendas have recognised disparities in health outcomes and emphasised the need to ensure universal health coverage (UHC). The Alma-Ata conference in 1978 prominently set the stage for health policy reforms globally―espousing primary health care (PHC) as an approach to improving health and well-being (Hall and Taylor, 2003; World Health Organization, 2008). PHC is defined as ‘essential health care based on practical, scientifically sound and socially acceptable methods and technology, made universally accessible to individuals and families in the community through their full participation and at a cost that the community and the country can afford to maintain at every stage of development in the spirit of self-reliance and self determination’ (World Health Organization, 2008: 5). Thus, PHC is considered a useful approach that seeks to address health and health care inequalities through the provision of basic but comprehensive and affordable health care services (Hall and Taylor, 2003).

Despite these efforts, health care access trends in low- and middle-income countries persistently reveal wide inequalities (Gwatkin *et al.*, 2007; World Health Organization, 2016). Sub-populations with the worse health outcomes include women, rural residents and the poor (World Health Organization, 2016). To this end, the recently endorsed Sustainable Development Goals (SDG), inspired by progress in health care access, seek to ensure healthy lives and well-being for all – with emphasis on UHC, access to quality essential care, financial support, safe, quality and affordable medicines and vaccines by 2030 (World Health Organization, 2016). In Ghana, the Community-based Health Planning and Services (CHPS) is a PHC policy implemented alongside other health equity policies including the pro-poor national health insurance programme and the free maternal health care services. All these efforts recognise the need for bridging gaps in health and health care disparities.

Similar to many low- and middle-income countries, evidences reveal geographical and socio-economic disparities in health and health care service use in Ghana (Adongo *et al.*, 2013; Ghana Statistical Service, 2014b; World Health Organization, 2015). Access to health care among women is low relative to their health needs (World Health Organization, 2015; Ghana Health Service, 2015b;). Women in rural localities are observed to face enormous barriers in accessing health care, including poor transportation networks, longer distance to health facilities, inadequate health care professionals, lack of logistics and abusive and non-receptive attitude of health care professionals (Atuoye *et al.*, 2015; Rishworth *et al.*, 2016). Also, a study by Yakong *et al.* (2010) in rural Ghana revealed unethical practices by health care professionals such as intimidation and limited sources of health care as factors hindering women’s uptake of facility delivery services. As we prepare to revisit the post Alma-Ata declaration on PHC with impetus from the Tokyo declaration of UHC, it is crucial that we take stock of the impact of health equity policies and the remaining gaps in health care access in low- and middle-income countries. In this regard, we examine the contribution of Ghana’s CHPS policy to women’s access to health care services.

## The Community-based Health Planning and Services (CHPS) policy in Ghana

In addressing the challenges to health care access, the government of Ghana through the Ghana Health Service in 1999 started the implementation of the CHPS (Nyonator *et al.*, 2005; Atuoye *et al.*, 2015). CHPS is a PHC model that seeks to enhance UHC by providing basic and affordable health care services (e.g., treatment of minor illnesses, antenatal services, health education and community sensitisation) to people (Nyonator *et al.*, 2005). CHPS operates on the principle of community’s ownership of health and health care delivery. It involves community resource mobilisation, community volunteerism and engagement with a resident community health officer (CHO). Health care services are provided at the CHPS compound, which also serves as residence for the CHO. The CHOs work with trained community health volunteers (CHVs) in basic health surveillances, health promotion and health education. Health conditions beyond the capacity of CHPS are referred to higher-level health facilities, including clinics, polyclinics and hospitals. Since its adoption, the policy expanded to cover many rural areas and deprived urban localities with 5421 functional CHPS compounds in 2016 (Ghana Health Service, 2018).

Despite empirical studies suggesting that CHPS could be improving women’s access to PHC, widening health care access disparities continue to be reported in the country, raising questions about the extent to which the policy is achieving its goals (World Health Organization, 2015; Ghana Health Service, 2015a;). As intimated by Goddard (2009), health care access policies are effective only when the targeted barriers to access have been eliminated. Commonly cited challenges impeding the effectiveness of health policies in low- and middle-income countries include funding constraints, weak institutions, inadequate commitment of stakeholders and the overambitious nature of these policies (World Health Organization, 2015). As most of these known challenges are addressed in the unique design of CHPS, it has been suggested that the policy could significantly contribute to reducing health care access inequalities. Yet, there is limited understanding of the contribution of CHPS to PHC access in the country. Most studies on health care access in the context of CHPS have centred on the general population, and the few specific to women focus on maternal health care services (Atuoye *et al.*, 2015; Shamsu-Deen, 2015; Woods *et al.*, 2018). This study examines the contribution of CHPS to PHC services access among women in the Upper West Region (UWR) of Ghana by hypothesising that women in CHPS zones (mostly rural areas) are more likely to access the needed health care than their counterparts in non-CHPS zones.

## Theoretical background

Cognisant of the myriad of factors influencing women’s use of health care services and the complex relationship between them (Fotso *et al.*, 2009; Ganle *et al.*, 2015), this study draws on Peters *et al.* (2008) framework for assessing access to health care services to understand the impact of the CHPS policy on women’s access to health care services in the UWR of Ghana. Peters *et al.* (2008) model identifies four main dimensions to be dictating health care service use―geographical accessibility, availability, financial accessibility and acceptability. Geographical accessibility denotes proximity of health facility/services to users, manifested in their place of residence and distance to health facilities. Aimed at eliminating these geographical barriers, it is expected that residence in a CHPS zone will enhance access to care.

Availability is a supply-side dimension that relates to one’s ability to access the right type of service during need, measured in terms of operational hours, availability of health professionals and logistics (Goddard and Smith, 2001). Indeed, as the first point of contact, CHOs reside in these facilities and provide services throughout the day. Outreach services are provided to communities and homes within the zone to further ensure service availability. Financial accessibility, on the other hand, involves consumers’ ability and willingness to pay for services rendered them, and this is often measured using wealth. In response to financial barriers to health care service use, CHOs in collaboration with CHVs are required to embark on initiatives such as community emergency transport services which will help reduce the financial burden of accessing health care and improve people’s uptake of health care service (Woods *et al.*, 2018). This is particularly pertinent to the context of the UWR, which is characterised by high poverty rates (Ghana Statistical Service, Ghana Health Service and ICF International, 2015). Lastly and critical to the success of any health system is service acceptability, which is a function of the fit between provided service and user expectations. While education, age and religion have been identified as important correlates of health care service use, they have also been reported to greatly influence service acceptability (Peters *et al.*, 2008; Negin *et al.*, 2009; Sano *et al.*, 2018a).

These dynamics should be analysed with the understanding that gender-based inequalities shape health-seeking behaviours (Sen and Östlin, 2008). In the UWR, where social norms perpetuate male dominance, women’s access to household resources and personal agency may be hampered which has implications for their health choices (Ghana Statistical Service, 2013; Atuoye *et al.*, 2015). As noted by Shaikh *et al.* (2008), decision-making among married women leads to delays in seeking care. Indeed, situating this study within the context of social inequalities will aid in understanding how the CHPS initiative and women’s socio-economic and demographic characteristics collectively influences their access to health care in the UWR of Ghana.

## Study area

The UWR is located in north-western Ghana and is largely rural (83.7%) with a population of 702,110 (Ghana Statistical Service, Ghana Health Service and ICF International, 2015). The sex distribution of the region is relatively uniform with females constituting 51.4% of the population. It is the poorest region in the country with a 70.7% poverty incidence and with over two-thirds of households income coming from agriculture (Ghana Statistical Service, Ghana Health Service and ICF International, 2015). In Ghana, the poor are classified as people whose annual income is below 1,314 Ghana cedis. The region is dominated by a patriarchal social system with its attendant effects on women’s autonomy and control over the already-scarce household resources. Christianity (44.5%) is the dominant religion, followed by Islam (35.6%) and African traditional religions (13.9%) (Ghana Statistical Service, 2013). There exist high illiteracy rates in the region with about 48.7% of women aged 15–49 having no form of formal education (Ghana Statistical Service (2015); Ghana Statistical Service, Ghana Health Service and ICF International, 2015).

As part of efforts to improve PHC in Ghana, 256 CHPS zones had been created as of 2017 (Ghana Health Service, 2018). Despite this, overall access to health care among women is low with some progress in certain aspects of maternal health care. While the region has an antenatal care coverage of 85.2%, only 72.3% meet the World Health Organisation–recommended 4+ visits (Ghana Health Service, 2015a) and may be much lower in very remote areas. Also, many deliveries still take place unassisted, leaving assisted/facility delivery rate at 63.9% in 2014 (Ghana Health Service, 2015a). However, there is a reported 93.7% postnatal services coverage in the region (Ghana Statistical Service, 2014b).

## Methods

### Sampling and data collection

A cross-sectional survey involving 805 women was conducted in seven districts in the UWR. Sampling involved a multi-stage sampling technique. In the first stage, the Ghana Living Standards Survey Round 6 (GLSS 6) reported poverty index was used to randomly select seven districts, comprising three from the rich districts cluster and four from poor districts cluster (Ghana Statistical Service, Ghana Health Service and ICF International, 2015). This was done to ensure wealth variation in the districts that formed the analytical sample. Subsequently, participants were selected proportionally to the total population of the districts using the 2010 Population and Housing Census figures (Ghana Statistical Service, 2013). For each district, approximately 70% of study participants were drawn from CHPS zones and the remaining from non-CHPS zones to reflect CHPS coverage in the region in 2015 when the data were being collected. Finally, a systematic random sampling technique was used to select every fifth household, starting from one end of the community. A woman from each selected household with the delivery date closest to the data collection date was interviewed using a structured questionnaire modified from the GLSS 6 instrument for the purposes of the study. We excluded households without women who had delivered in the last five years preceding the study to reduce the effect of recall bias. Ethical approval for the study was obtained from the University of Western Ontario Non-Medical Research Ethics Board and all protocols duly adhered to. Data were collected on health care access, presence of CHPS, distance to health facility, household assets and the demographic and socio-economic characteristics of participants.

### Measures

With the aim to understand women’s access to health care within the context of CHPS, participants were asked the question; ‘were you able to seek treatment from a health care provider when you needed care?’ This question was adopted as a measure of the independent variable because both the previous Millennium Development Goals and the current SDGs target universal access to health care, epitomised in the ability to seek care from a professional when in need. Kuuire *et al.* (2015) in assessing health-seeking behaviour among adults during illness in Ghana used a similar question as a measure of health care access. Also, Farrants *et al.* (2017) and Porell and Miltiades (2001) used a similar question to assess health care access in their studies. Access to professional care is essential given that varied sources of care, many of which are inappropriate, such as the traditional healers and drug dispensing kiosks exist in this context. Women’s access to care from a health professional was treated as a binary variable with the inability to access care coded as ‘0’ and the ability to access care coded as ‘1’. The focal independent variable is whether respondents live in a CHPS zone (0 = no; 1 = yes). In addition, three sets of variables were controlled for, namely, locational, socio-economic and demographic variables. Locational variables include urban-rural residence and distance to health facility. For socio-economic variables, we included household wealth quintiles and level of education. Household wealth quintile was constructed using a composite index of household assets including annual income, electricity, drinking water, radio set, television set, car, motor, toilet facility and livestock (Gwatkin *et al.*, 2007). Finally, we controlled for three demographic variables, namely, age of respondents (measured in completed years), marital status and religion.

### Analysis

Given that the dependent variable is dichotomous, the logit regression technique was employed to explore the relationship between the dependent and independent variables. The analysis was carried out using STATA 14 software. Three multivariate models (Models 1–3) were built to examine the effect of CHPS on the ability of women to access health care services when needed. Model 1 included residence in CHPS zone and two other locational variables (rural-urban residence and distance to health facility), with socio-economic and demographic variables controlled for in Models 2 and 3, respectively. For meaningful interpretation, findings have been reported in odds ratios (OR). OR greater than one indicates that women are more likely to seek treatment from health care providers, while those less than one implies a lesser likelihood of doing so.

## Results

### Univariate results

Majority of women (83.60%) reported that they were not able to seek care from a health care worker when they needed it, even though a high proportion of them resided in CHPS zones (76.77%) (Table 1). While household wealth quintiles were relatively equally distributed, more than half of respondents (57.02%) reported that they did not have any formal education. Also, the largest proportion of women were Christians (49.94%), followed by Muslims (39.50%) and Traditionalists (8.70%).

**Table 1.** Univariate analysis of selected dependent and independent variables

Variable	Category	Codes^a^	Percentage
*Accessed treatment from a health worker*			
	No	0	83.60
	Yes	1	16.40
*Residence in CHPS zone*			
	No	0	23.23
	Yes	1	76.77
*Urban-rural residence*			
	Urban	0	27.20
	Rural	1	72.80
*Distance to health facility*	Less than 1 km	0	38.39
	From 1 to 5 km	1	24.35
	More than 5 km	2	37.27
*Household wealth quintile*			
	Richest	0	19.25
	Richer	1	18.39
	Middle	2	18.51
	Poorer	3	23.48
	Poorest	4	20.37
*Level of education*			
	Secondary/higher	0	22.11
	Primary education	1	20.87
	No education	2	57.02
*Age of respondents*^b^	30.82		
*Marital status*			
	Married	0	83.23
	Never married	1	4.47
	Formerly married	2	12.30
*Religion*			
	Christian	0	49.94
	Muslim	1	39.50
	Traditionalist	2	8.70
	No religion/other	3	1.86
Total			805

a The categories coded ‘0’ are the reference categories for all variables.

b Mean reported for age of respondents.

### Bivariate results

Table 2 details results from the bivariate analysis. Women residing in CHPS zones reported having more access to care from a health care worker when they needed it relative to their counterparts in non-CHPS zones (OR = 1.426; *P ≤* 0.01). Compared to women who resided less than a kilometre (km) from the health facility, those who lived more than 5 km away were less likely to seek care from a health professional (OR = 0.851; *P ≤* 0.1). For household wealth quintiles, women from the poorer and poorest households were less likely to access care when compared with those from the richest households (OR = 0.673; *P ≤* 0.01 and OR = 0.805; *P ≤* 0.1, respectively). For demographic variables, older women were more likely to seek care from a health professional than their younger counterparts were (OR = 1.012; *P ≤* 0.01). Moreover, never married and formerly married women were both more likely to seek care from a health professional than currently married women were (OR = 2.503; *P ≤* 0.01 and OR = 1.607; *P ≤* 0.01, respectively).

### Multivariate results

For the multivariate analysis (see Table 3), three models were built to show the relationship between our independent variables and women’s access to health care. Findings were generally consistent with the bivariate results even after controlling for socio-economic and demographic variables. Women residing in CHPS zones were still more likely to seek treatment from a health care worker than those in non-CHPS zones (OR = 1.612; *p ≤* 0.01). Some control variables also remained statistically significant. For instance, compared with urban women, rural women were less likely to access health care (OR = 0.696; *P ≤* 0.05). Also, women who lived over 5 km away from the health facility were less likely to access health care compared to those who lived less than a kilometre away from a health facility.

**Table 2.** Bivariate analysis of selected dependent and independent variables

Variable	OR (SE)
*Residence in CHPS zone*	
No	1.000
Yes	1.426 (0.155)^***^
*Urban-rural residence*	
Urban	1.000
Rural	0.998 (0.099)
*Distance to health facility* Less than 1 km	1.000
From 1 to 5 km	0.923 (0.105)
More than 5 km	0.851 (0.086)*
*Household wealth quintile*	
Richest	1.000
Richer	0.929 (0.133)
Middle	0.925 (0.132)
Poorer	0.673 (0.093)^***^
Poorest	0.805 (0.113)^*^
*Level of education*	
Secondary/higher	1.000
Primary education	1.052 (0.142)
No education	1.056 (0.117)
*Age of respondents*	1.012 (0.005)^**^
*Marital status*	
Married	1.000
Never married	2.503 (0.588)^***^
Formerly married	1.607 (0.218)^***^
*Religion*	
Christian	1.000
Muslim	1.047 (0.098)
Traditionalist	0.847 (0.140)
No religion/other	1.673 (0.566)

OR = odds ratios; SE = standard errors.

*P ≤ 0.1; ^**^P ≤ 0.05; ^***^P ≤ 0.01.

**Table 3.** Multivariate analysis of ‘access to treatment from health care worker’ among women in the UWR, Ghana

Variable	Model 1	Model 2	Model 3
	OR (SE)	OR (SE)	OR (SE)
*Residence in CHPS zone*			
No	1.000	1.000	1.000
Yes	1.472 (0.167)^***^	1.580 (0.180)^***^	1.612 (0.196)^***^
*Urban-rural residence*			
Urban	1.000	1.000	1.000
Rural	0.929 (0.096)	0.767 (0.099)^**^	0.696 (0.099)^**^
*Distance to health facilities*			
Less than 1 km	1.000	1.000	1.000
From 1 to 5 km	0.972 (0.112)	0.923 (0.115)	0.885 (0.113)
More than 5 km	0.845 (0.089)*	0.805 (0.088)^**^	0.809 (0.091)^*^
*Household wealth quintiles*			
Richest		1.000	1.000
Richer		0.904 (0.132)	0.926 (0.141)
Middle		0.861 (0.126)	0.863 (0.134)
Poorer		0.580 (0.084)^***^	0.581 (0.091)^***^
Poorest		0.644 (0.112)^**^	0.608 (0.110)^***^
*Level of education*			
Secondary/higher		1.000	1.000
Primary education		1.007 (0.143)	1.095 (0.162)
No education		0.906 (0.116)	0.973 (0.136)
*Age of respondents*			1.006 (0.006)
*Marital status*			
Currently married			1.000
Never married			2.825 (0.707)^***^
Formerly married			1.553 (0.242)^***^
*Religion*			
Christian			1.000
Muslim			1.012 (0.110)
Traditionalist			0.744 (0.142)
No religion/other			1.438 (0.531)
Constant	0.460 (0.565)^***^	0.698 (0.148)^*^	0.526 (0.151)^**^
Log likelihood	−352.146	−343.754	−324.93

OR = odds ratios; SE = standard errors; locational variables in Model 1; socio-economic variables added in Model 2; demographic variables in Model 3.

*P ≤ 0.1; ^**^P ≤ 0.05; ^***^P ≤ 0.01.

With regard to the socio-economic and demographic variables, household wealth and marital status of women remained robustly associated with their access to health care. In comparison to richest households, poorer and poorest households were less likely to have access to care (OR = 0.581; *P ≤* 0.01 and OR = 0.608; *P ≤* 0.01, respectively). Contrary to the bivariate results, women’s age became insignificant after controlling for socio-economic variables. Furthermore, never married and formerly married women were more likely to have access to health care compared to their married counterparts (OR = 2.825; *P ≤* 0.01 and OR = 1.553; *P ≤* 0.01, respectively).

## Discussion

We examined the relationship between CHPS, a PHC promotion policy and women’s access to basic health care services in the UWR of Ghana. Overall, our findings reveal increased access to health care service among women residents in CHPS zones. With the goal to contribute to community health and sustainability, our findings suggest the policy may be an effective initiative in this regard and efforts at ensuring UHC in the country. In the midst of ubiquitous unmet health needs, particularly among the less privileged, this study contributes to the literature by highlighting how structural- and individual-level factors intersect to shape the utilisation of health care services. This has implications for health policy formulation and implementation in Ghana and similar contexts.

The focus of any health care policy including the CHPS policy is to enhance geographical accessibility to health care services. This is because geographical barriers have been widely shown to hinder access to health care, especially for rural dwellers (Gabrysch and Campbell, 2009; Ntsua *et al.*, 2012; Atuoye *et al.*, 2015). Consistent with previous studies, our findings reveal improved access to care with residence in a CHPS zone (Johnson *et al.*, 2015; Woods *et al.*, 2018). Modelled around the zoning system, residing within the CHPS zone remarkably reduces the geographical distance previously covered to access health care services. Besides, CHOs and CHVs ensure health service availability by providing care all day as well as outreach services within the zone to meet the care needs of people who may still be physically restricted from accessing health care. Beyond this, the disproportionate burden of non-CHPS zone resident women in navigating social norms that restrict their movement in the region such as seeking for permission and performing domestic duties may be plausible for explaining this variation. For women living in CHPS zones, exposure to various resource mobilisation initiatives in addition to empowerment and capacity building programmes by CHOs and CHVs has positive implications for their access to care (especially reduction in financial burden of health care). Indeed, while acknowledging the contribution of the zoning system in enhancing geographic accessibility to care, our findings suggest that it may be biased towards women residing in CHPS zones, hence the need to scale up or redesign the model to cover many more communities, including those in deprived urban contexts.

Furthermore, an understanding of the role of CHPS in health care service accessibility ought to be within the context of inherent urban bias in infrastructural development in Ghana and other low- and middle-income countries that deprive rural dwellers access to health care services. Consistent with previous research (Osubor *et al.*, 2006; Ntsua *et al.*, 2012), we found that rural women were less likely to report having access to health care services compared to their urban counterparts. Rural-urban health care access disparities largely stem from the inadequacy of health facilities in rural areas, as well as difficulties and cost involved in navigating rural geographies to access care (Atuoye *et al.*, 2015). Besides, rural women compared to their urban counterparts may be more likely to uphold and adhere to traditional norms that perpetuate male dominance and social inequalities, which have deleterious implications for their health care–seeking behaviours. Unfortunately, resource constraints and the geographies of rural Ghana largely limit the capacity to increase CHPS coverage to many deprived settings.

Even though part of the CHPS mandate is to reduce geographic distances to health facilities, longer distances to these facilities still persist as a disincentive to women seeking health care, and this would likely be exacerbated by the poor transport and road networks that characterise many rural areas in the country (Shaikh and Hatcher, 2005; Ghana Statistical Service, 2014a; Atuoye *et al.*, 2015). Consistent with our finding, Kuuire *et al.* (2015) in examining health care–seeking behaviour in resource poor settings in Ghana assert that longer distances to health facilities hinder uptake of professional care. Indeed, most roads in rural Ghana are not motorable especially during the rainy seasons. While this may not be a challenge to women living closer to health facilities, their counterparts who live farther away from health facilities may be overburdened. They may have to either walk longer or seek assistance from male family members to get to these facilities (Atuoye *et al.*, 2015; Rishworth *et al.*, 2016). Moreover, seeking permission and/or financial support to travel to these distant facilities may be more challenging for women, possibly accounting for the variations in access. In overcoming transportation barriers to accessing health care services, Woods *et al.* (2018) notes the emergence of the community emergency transport systems (CETS) in some CHPS operational communities. CETS are local-level transport arrangements to assist patients to seek care at referral points in a timely manner in the absence of a working ambulance system for rural communities. Indeed, this community-based innovation has been touted as one key strategy of increasing access to critical health care, including obstetric care for women in deprived communities.

We found a significant association between household wealth and health care access with poor women less likely to access health care services compared to their richer counterparts. Financial inaccessibility manifested in facility user fees and cost involved in acquiring medications have been widely acknowledged to hinder access to care in low- and middle-income countries (O’Donnell, 2007; Anfaara *et al.*, 2018). Despite reports that the Ghana National Health Insurance Scheme and the maternal exemption policy under the scheme have improved women’s access to health care, poor women may still be challenged in paying insurance premiums and renewals, transportation cost to health facilities, as well as user and prescription cost not covered by these pro-poor policies (Dixon *et al.*, 2014; Atuoye *et al.*, 2016).

In addition, we found that marital status is associated with women’s access to health care in the UWR of Ghana. Disparities in levels of autonomy and personal agency in this predominantly patriarchal society could explain this finding (Sano *et al.*, 2018b). Thus, despite the support married women may receive from their partners, the decision and means to access health care services still lies in the hands of male partners, household heads and older women (e.g., mothers in-law) in this context (Ganle *et al.*, 2015). Unmarried women relative to their married counterparts are more likely to be mobile, easy to mobilise and have more control over their health choices. As noted by Shaikh *et al.* (2008), health care decision-making among partnered women causes delays in seeking care. Moreover, with control over their lives, unmarried women may be more receptive to capacity building and empowerment programmes by CHOs and CHVs. Our finding suggests that cultural processes perpetuating male dominance have adverse implications for the CHPS policy in the region.

In spite of the importance of our study in situating women’s health care service access within the context of the CHPS model, there are some limitations largely arising from the nature of the study design worth noting. First, the study is cross-sectional which limits the interpretation of the findings to associations between the explanatory and response variables. It does not infer cause and effect. Second, the study relied on self-reported health care access, which may be affected by recall bias. This study has not also been able to account for all variables cited within the literature to be influencing health care service use among women. Nonetheless, these findings are generally consistent with the literature on health care access in low- and middle-income countries.

## Conclusion

This paper examined the role of the CHPS policy on women’s access to health care services in the UWR of Ghana. Consistent with previous studies, findings show that CHPS has enhanced the access to basic health care services in the region. Analysis of the potential for CHPS in promoting health care access is used to highlight progress made on PHC and UHC fronts, and current gaps/barriers to health care access persistent in low- and middle-income countries. An understanding of how the CHPS policy intersects with people’s socio-demographic and economic characteristics to influence their health-seeking behaviours is essential for the design and implementation of efficient and sustainable PHC policies. The study recommends that the number and quality of CHPS compounds should be increased to provide easy access to quality care. In addition, health service providers (CHOs and CHVs) should be supported, incentivised and offered training/retraining to keep abreast with health issues. There is also the need to develop and engage in gender-sensitive programmes that will help build the capacities of women within the households to seek health care services.
